# 

**Published:** 1879-05

**Authors:** 


The Session of the American Medical Association opens at 10 A. M., Tuesday, May 6th, at Atlanta, Ga., and continues four days. An interesting and instructive meeting may be expected. The National Medical Conventions that gather at Atlanta in May, open by the Convention of Medical Colleges that is called to assemble in the State Senate Chamber of that city, Friday morning, May 2, at 10 A. M. This is followed by the third annual meeting of the American Medical College Association at the same place, at 10 A. M., Saturday, May 3. The Convention of American Medical Editors meets at 8 A. M on Monday, May 5.

### Books Received.

---

An Atlas of Human Anatomy, illustrating most of the Ordinary Dissections, and many not usually practised by the Student, accompanied by an Explanatory Text. By Rickman John Godlee, M. S., F. R. C. S., Fellow of University College, &c. Philadelphia: Lindsay & Blackiston.

Essays on Surgical Anatomy and Surgery: Comprising an Essay upon the Surgical Anatomy of the Common, External and Internal Carotid Arteries; an Essay upon the Surgical Anatomy and History of the Innominate and

Subclairon Arteries; an Essay upon the Surgical Anatomy of the Tibio-Tarsal region; an Essay upon the Surgical Anatomy of the Ablurator Artery, and Notes upon the Surgical Anatomy of the Hip-joint. By John A. Wyeth, M. D., (University of Louisville). New York: Wm. Wood & Co. Buffalo; J. H. Matteson. pp. 262.

Diphtheria; its Nature and Treatment. Mackenzie. Philadelphia: Lindsay & Blackiston.

A Practical Treatise on Surgical Diagnosis. Designed as a Manual for Practitioners and Students. By Ambrose L. Ranney, A. M., Adjunct Professor of Anatomy and Lecturer on Minor Surgery in the Medical Department of the University of New York. New York: Wm. Wood & Co: Buffalo: Peter Paul & Bro. pp. 375.

The Achievements of Stanley and other African Explorers. By Hon. J. T. Headley. Illustrated. Philadelphia: Hubbard Bros. pp. 605.

A Guide to the Qualitative and Quantitative Analysis of the Urine. Designed for Physicians, Chemists and Pharmacists. By Dr. Neubauer, Professor, Chief of the Agricultural-Chemical Laboratory and Docent in the Chemical Laboratory-in Wiesbaden, and Dr. J. Vogel, Professor of Medicine in the University at Halle; with a Preface by Prof. Dr. Fresenius. Translated from the Seventh Enlarged and Revised German Edition. By Elbridge G. Cutter, M. D., Physician to Out Patients at the Massachusetts General Hospital, Pathologist at the Boston City Hospital, and Assistant in Pathology in the Medical School of Howard University. Revised by Edward S. Wood, M. D., Professor of Chemistry in the Medical School of Howard University. New York: Wm. Wood & Co. Buffalo: J. H. Matteson. pp. 551.

Chemistry: General, Medical and Pharmaceutical; including the Chemistry of the U. S. Pharmacopœia; A Manual on the General Principles of the Science and their Applicatiions in Medicine and Pharmacy. By John Attfield, M. A. and Ph. D of the University of Tübingen; Member of the Councils of the Institute of Chemistry of Great Britain and Ireland, and (lately) of the Chemical School of London, etc , etc. Eight Edition. Revised by the Author. Philadelphia: Henry C. Lea. Buffalo: T. H. Butler. pp. 687.

On Diseases of the Abdomen: comprising those of the Stomach and other parts of the Alimentary Canal, Æsophagus, Cæcum, Intestines and Peritoneum. By. S. O. Habershon, London; Fellow of the Royal College of Physicians, etc., etc., with Illustrations. Second American from the Third Enlarged and Revised English Edition. Philadelphia: Henry C. Lea. Buffalo: T. H. Butler. pp. 554.

Epitome of Skin Diseases, with Formulæ for Students and Practitioners. By Tilbury Fox, M. D., F. R. C. P., Physician to the Department of Skin Diseases in University College Hospital, and T. C. Fox, M. B. A. (Cantab.), Physician to Saint George's and Saint James Dispensary. Second American Edition. Philadelphia: Henry C. Lea. Buffalo: T. H. Butler. pp. 216.

A Clinical Treaties on Diseases of the Liver. By Dr. Fried. Theo. Frerichs, Professor of Clinical Medicine in the University of Berlin, &c.; in three volumes; Vol. 2. Translated by Charles Murchison, M. D., F. R. C. P., Physician to the London Fever Hospital, &c., &c. New York: Wm. Wood & Co. pp. 228.

The Principles and Practice of Gynæchology. By Thomas Addis Emmet, M. D., Surgeon to the Woman's Hospital of the State of New York, &c. With one hundred and thirty Illustrations. Philadelphia: Henry C. Lea. Buffalo: T. H. Butler. pp. 855.

A Manual of Examination of the Eyes. A Course of Lectures Delivered at the "E'Cole Pratique." By E. Landolt, Director-Adjoint of the Opthalmological Laboratory at the Sorbonne, Paris. Translated. By Swan M. Burnett. M. D., Lecturer on Opthalmology and Otology in the Medical Department of the University of Georgetown, &c. Philadelphia: D. C. Brinton. pp. 312.

A Treatise on the Diseases of Infancy and Childhood. By J. Lewis Smith, M. D., Clinical Professor of Diseases of Children in Bellevue Hospital Clinical College. With Illustrations. Philadelphia: Henry C. Lea. Buffalo: Theo. H. Butler. pp. 758.

———:o:———

### Pamphlets Received.

Conclusions from the study of one hundred and twenty-five cases of Writer's Cramp and allied affections. By George M. Beard, M. D., New York.

On the Permanent Removal of the Hair by Electricity. By George Henry Fox, A. M., M. D. New York.

Opthalmia Neonatorum. By Richard H. Lewis, M. D. Ralegh, N. C.

How shall the Degree of Doctor of Medicine be Conferred ? By E. Fletcher Ingalls, M. D., Rush Medical College, Chicago, Ill.

Ringworm in Public Institutions. By John V. Shoemaker, A. M. Philadelphia, Pa.

Valedictory Address to the Graduating Class of Jefferson Medical College, at the forty-fourth Annual Commencement. By J. Aitken Meigs, M. D. Philadelphia, Pa.

Opium as a Tonic and Alterative; and Hypodermic Use, in the Debility and Amanrosis, sometimes consequent upon Onanism. By B. A. Pope, M. D. New Orleans.

Transactions of the Detroit Medical and Library Association. April, 1876.

Circulars for Information of the Bureau of Education. Nov. 1, 1879. Training Schools for Nurses.

Supplementary Rectal Alimentation, and especially by Defibrinated Blood, as applicable to a large range of cases in which Nutritive Enemata have not heretofore been employed. By Andrew H. Smith, M. D., Physician to St. Luke's Hospital, New York.

Ang (3 cop

### THE BUFFALO

### MEDICAL AND SURGICAL

### MEDICAL AND SURGICAL JOURNAL.

PUBLISHED MONTHLY.—Containing Original Articles, Reports of Medical Societies and Hospitals, Editorial, Reviews, Correspondence, Army News, etc., etc; including the usual variety of Medical Periodical Publications. Specimen copies sent on application to BUFFALO MEDICAL AND SURGICAL JOURNAL, J. F MINER, M. D., 978 Main Street, BUFFALO, N. Y.

TERMS OF SUBSCRIPTION, $3 00 PER YEAR, IN ADVANCE,—All communications for publication should be sent before the fifteenth (15) of each month, or they will of necessity lie over until the next number. No change can be made in advertisements after the twenty-fifth.

Correspondence and original articles are solicited. Publication is no evidence that the view of the author are endorsed by the Journal.

